# Supplementary material for: Identification of Epigenetic Mechanisms Involved in the Anti-Asthmatic Effects of Descurainia sophia Seed Extract Based on a Multi-Omics Approach
Source: Molecules. 2018 Nov 5;23(11):2879. doi: 10.3390/molecules23112879 (PMC6278437; doi:10.3390/molecules23112879)
Supplement: Supplementary file 1 [file molecules-23-02879-s001.zip › molecules-380405-SM/molecules-380405-SM.pdf]

Identification of epigenetic mechanisms involved in the anti-asthmatic effects of *Descurainia sophia* seed extract based on a multi-omics approach

Su-Jin Baek, Jin Mi Chun, Tae-Wook Kang, Yun-Soo Seo, Sung-Bae Kim, BoSeok Seong, Yunji Jang, Ga-Hee Shin, Chul Kim*

Supplementary information:

1. Supplementary Table (7) - Excel
   1.1. Table S1. DMR list in OVA vs. Saline
   1.2. Table S2. DMR list in DSE vs. OVA
   1.3. Table S3 DMR list in Anti-asthmatic patterns
   1.4. Table S4. DAVID result in hyper_hypo pattern
   1.5. Table S5. DAVID result in hypo_hyper pattern
   1.6. Table S6. DAVID result in 18 candidate genes
   1.7. Table S7. Network genes and chemicals associated with asthma
2. Supplementary Figure (5)
   2.1. Figure S1. Workflow of integrated profiling
   2.2. Figure S2. Experimental scheme and sample information
   2.3. Figure S3. Histogram for percent methylation distribution
   2.4. Figure S4. Histogram for the read coverage
   2.5. Figure S5. Correlation plot between methylation and expression of candidate genes

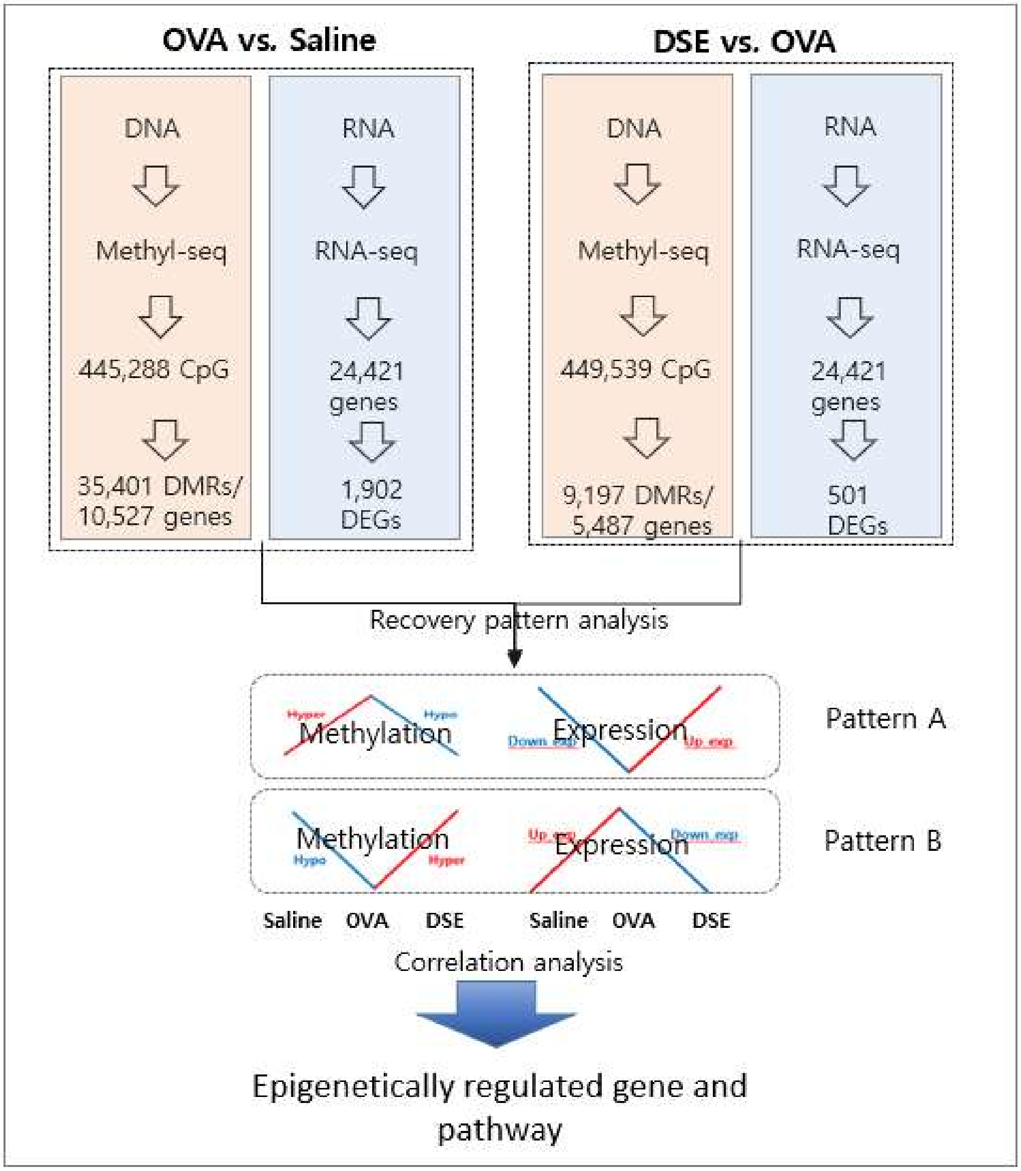

Figure S1. Workflow of integrated profiling Diagram describes integrated profiling process for identifying differentially methylated and expressed genes in saline, OVA, and DSE samples.

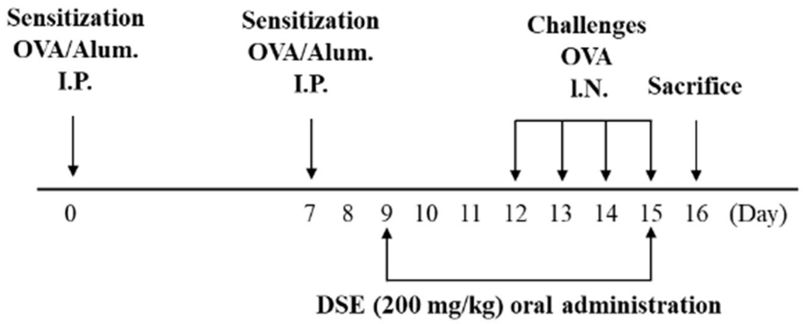

	Saline control	OVA induced	DSE treatment
Methyl-seq (n=10)	3	3	4
RNA-seq (n=10)	3	3	4

Figure S2. Experimental scheme and sample information. Schematic diagram depicts induction of airway inflammation in the mouse model. The table displays sample information for identifying DNA methylation and gene expression changes among the saline, OVA and DSE groups. Saline: control group, OVA: ovalbumin-induced group, DSE: *Descurainia sophia* seed extract group.

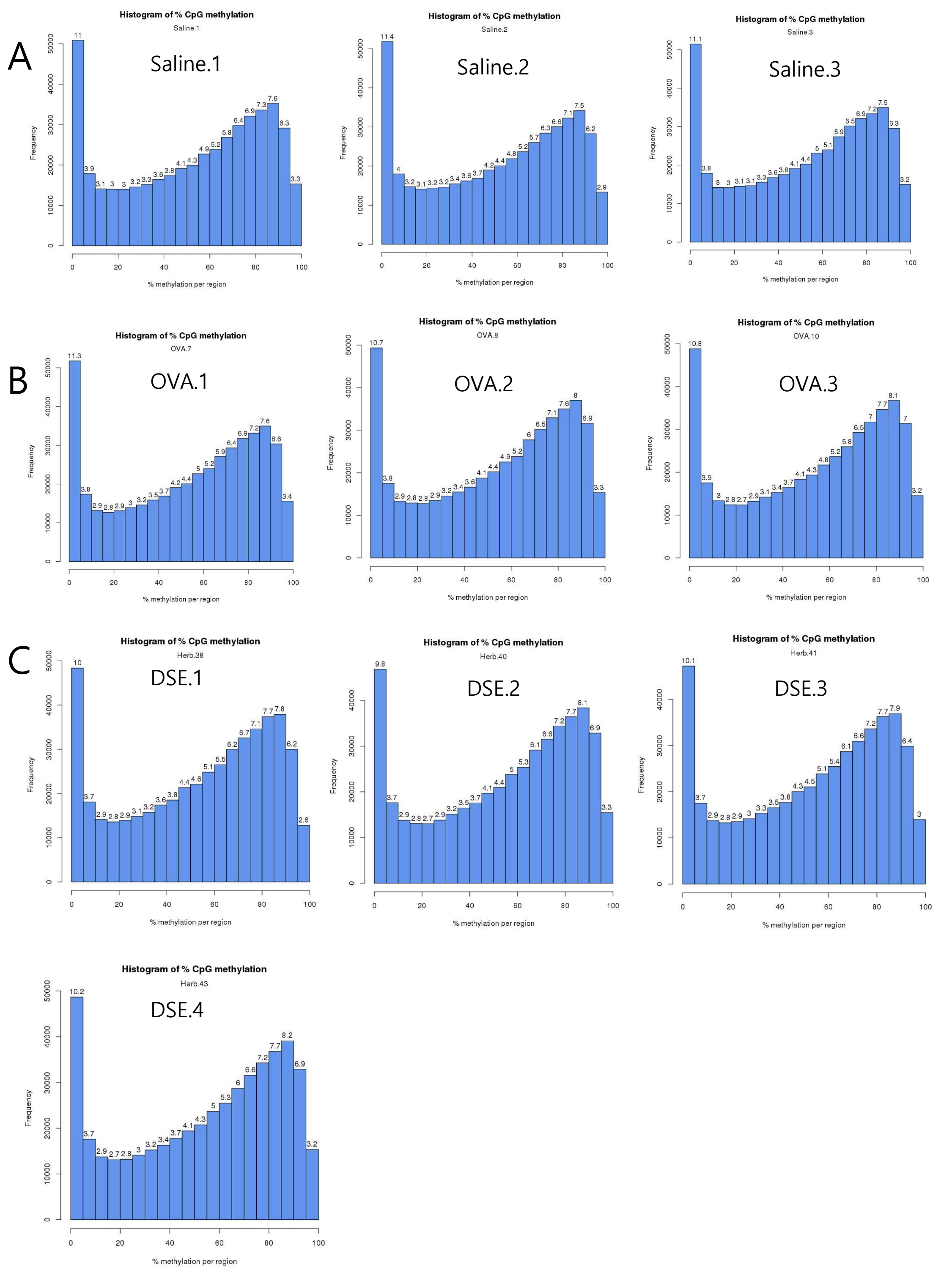

Figure S3. Histogram for percent methylation distribution. Numbers on bars denote what percentage of locations are contained in that bin in saline (A), OVA (B), and DSE (C) samples.

A Saline.1 Saline.2 Saline.3

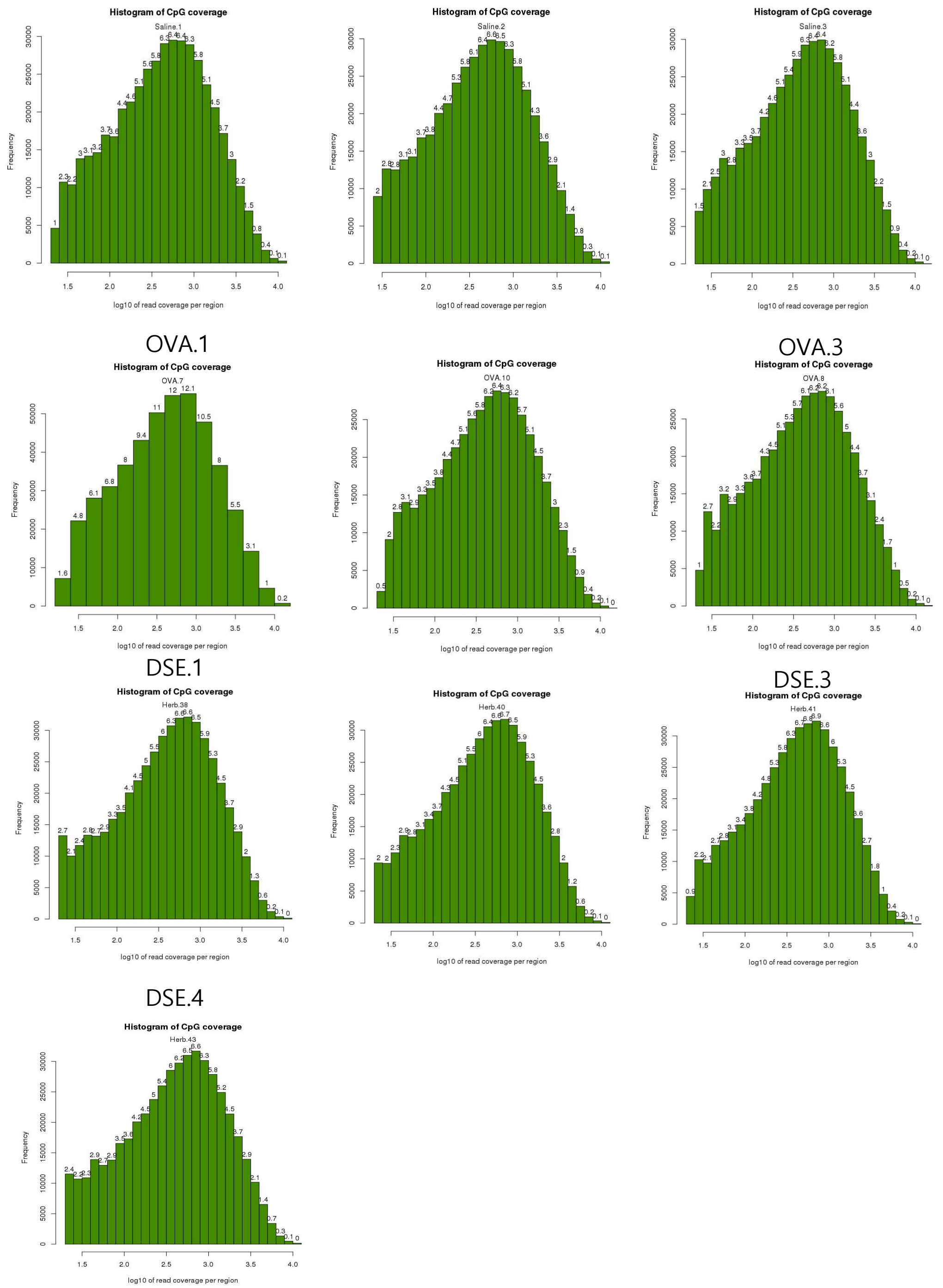

Figure S4. Histogram for the read coverage. Numbers on bars denote what percentage of locations are contained in that bin in saline (A), OVA (B), and DSE (C) samples.

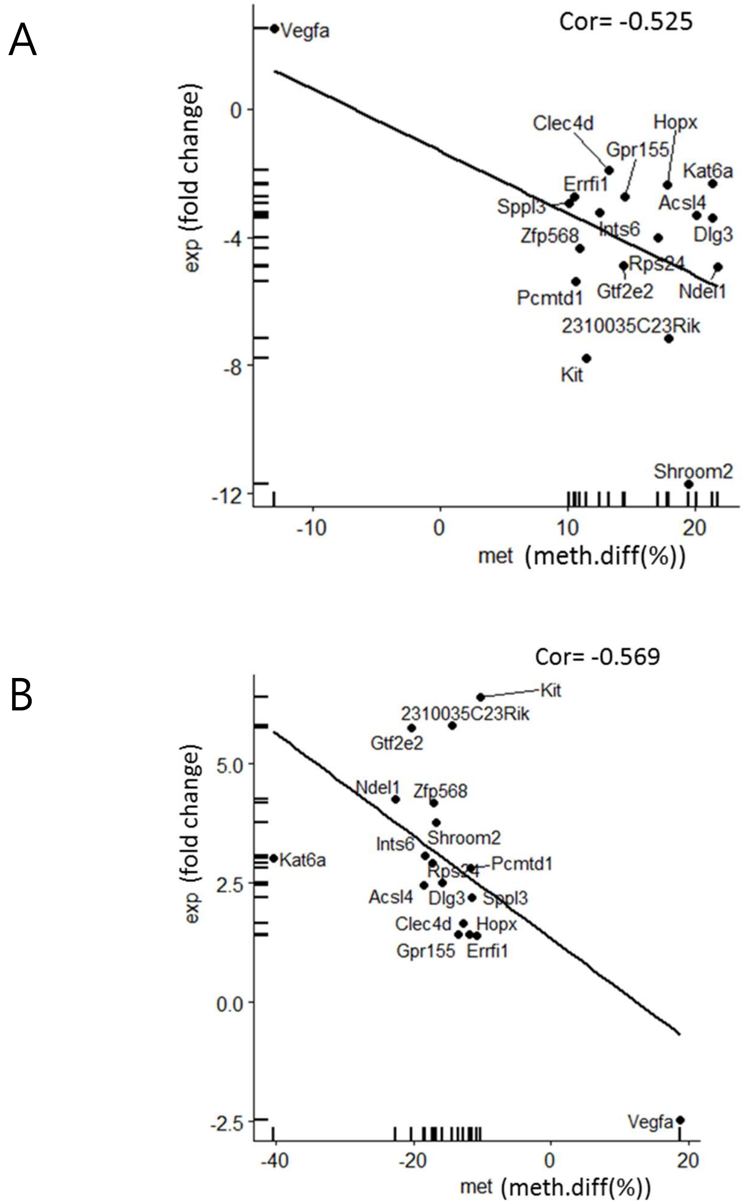

Figure S5. Correlation plot between methylation and expression of candidate genes. Comparison of DNA methylation and expression change in OVA vs. saline (A). Comparison of the DNA methylation and expression change in DSE vs. OVA (B).
